# Independent validation of time to treatment as a prognostic factor in uveal melanoma

**DOI:** 10.1186/s12885-026-15775-z

**Published:** 2026-02-19

**Authors:** Annahita Moghadam, Viktor Torgny Gill, Shiva Sabazade, Anna Hagström, Gustav Stålhammar

**Affiliations:** 1 Division of Eye and Vision, Department of Clinical Neuroscience, Karolinska Institutet, Eugeniavägen 12, Stockholm, 17164 Sweden; 2 Department of Pathology, Västmanland Hospital Västerås, Västerås, Sweden; 3 St. Erik Eye Hospital, Stockholm, Sweden

**Keywords:** Uveal melanoma, time to treatment, treatment delay, competing risks, Fine–Gray regression, prognosis

## Abstract

**Background:**

An earlier study suggested that longer intervals between diagnosis and treatment of uveal melanoma are associated with worse prognosis. We evaluated whether a similar association could be observed in an independent cohort.

**Methods:**

Retrospective cohort of 336 patients with posterior uveal melanoma treated at a single referral center (2000–2011), assembled by investigators independent of the original report. We compared disease-specific (DSS) and overall survival (OS) across prespecified diagnosis-to-treatment intervals (≤ 15, 30, 45 days) using cumulative-incidence (Gray’s) and Kaplan–Meier analyses (unmatched and propensity-matched). Fine–Gray regression with treatment day as time zero estimated the subdistribution hazard ratio per 10-day delay.

**Results:**

Tumor size and stage at diagnosis did not differ between patients treated within 30 days (prompt) and those treated after 30 days (delayed). In competing-risk analysis, delayed treatment was associated with a higher cumulative incidence of metastatic death. Kaplan–Meier OS—but not DSS—differed significantly between prompt and delayed treatment. A model of exponential tumor growth indicated that deferring treatment of a lesion of a medium-sized tumor by one month is associated with an estimated absolute increase in the 10-year competing-risk incidence of metastatic death by ≈ 0.8%.

**Conclusions:**

While treatment delay is unlikely to be among the strongest prognostic factors in uveal melanoma, this independent validation cohort supports an association between diagnosis-to-treatment intervals beyond about one month and worse survival.

**Supplementary Information:**

The online version contains supplementary material available at 10.1186/s12885-026-15775-z.

## Introduction

Uveal melanoma is the most common primary intra-ocular malignancy in adults, yet its management remains challenging. Despite recent advances in systemic therapy for metastatic disease, median survival after the onset of metastasis is still under two years [1–3]. 

At the heart of this challenge lies the ambiguous prognostic significance of the timing of primary tumor treatment. While numerous tumor characteristics have been associated with an increased risk of metastasis, the role of the timing of treatment remains debated [4–8]. On one side, effective treatments can prevent the tumor from increasing in size and spreading metastases—a crucial consideration given that larger tumors are often characterized by aggressive genetic and cytogenetic features [9–11]. Conversely, most authors argue that metastatic seeding occurs early in the disease course, often before the primary tumor is discovered and diagnosed [9, 12, 13]. Thus, if metastases that ultimately lead to mortality are presumed to be present before the eye receives treatment, the timing of such interventions might be relatively insignificant for patient survival. However, these two perspectives may represent a false dichotomy: Micrometastases may very well already be present when the primary tumor is detected, and obviously, we cannot prevent what has already happened, but prompt treatment upon discovery of a primary tumor may prevent further metastatic dissemination from a tumor that has grown larger and potentially acquired additional aggressive traits [14]. 

In a meta-analysis, we observed that among the rare cases in which treatment was deferred for more than five years after diagnosis, 88% (15 of 17) of those with small to medium-sized choroidal melanomas developed metastases during long-term follow-up [15]. In most cases, the long delays occurred because patients did not seem to understand the gravity of their disease or did not want to lose an eye. In some cases, the diagnosis was initially misinterpreted or neglected by the patient. Similarly, in a large consecutive cohort study, we demonstrated that even short intervals between diagnosis and treatment can adversely affect prognosis [16]. In that study, delays as brief as 30 days were associated with worse survival outcomes in American Joint Committee on Cancer (AJCC) stage II and III, but not I. This finding aligns with evidence from over a million patients across various cancer types showing the critical impact of treatment timing [17]. 

On the other hand, Damato et al. recently concluded that “Deferring treatment of choroidal melanomas until documentation of growth may delay iatrogenic visual loss by months or years and is associated with minimal increase in metastatic mortality.” [18] However, this interpretation warrants reevaluation: the 4.3% yearly growth rate reported by Damato et al. for 24 tumors corresponds to a volume doubling time of approximately 2018 days, assuming a semi-spheroidal shape with proportional increases in thickness and diameter—an exceptionally slow rate compared with findings from a recent meta-analysis, which showed an average volume doubling time of 360 days across all patients and 717 days for small melanomas [19]. In fact, if Damato et al. had applied growth rates more closely aligned with those from our meta-analysis, they would likely have reached conclusions about the prognostic impact of treatment delays that are consistent with Stålhammar [16]. 

It has been noted that in our previous work, the diagnosis-to-treatment interval—unknown at the time of diagnosis—was incorporated directly into regression models, and that the Fine–Gray approach did not consider treatment as a landmark event [16]. Further, although competing-risk analyses showed no difference in non-melanoma mortality between early- and delayed-treatment groups, residual imbalances in comorbidity could still have biased survival estimates.

The present study reassesses whether an association exists between treatment timing and prognosis in a non-overlapping cohort collected by investigators independent of the original work. We use propensity-score matching, set the day of treatment as time zero in competing-risk models, and test several thresholds defining early versus late treatment. This design aims to clarify the prognostic impact of treatment delay in uveal melanoma.

## Methods

### Aim of the study

To determine whether longer intervals between diagnosis and primary treatment increase the risk of death due to metastasis in posterior uveal melanoma.

### Study design and data sources

This retrospective cohort comprised all patients consecutively diagnosed with posterior uveal melanoma at St Erik Eye Hospital, Stockholm, between 1 January 2000 and 31 December 2011. Because digital medical records were not fully available before 2012, we combined electronic queries with manual review of (i) the hospital treatment registry and (ii) accession logs from the St Erik Ophthalmic Pathology Laboratory to retrieve earlier cases.

### Inclusion criteria

1. Posterior uveal melanoma involving the choroid and/or ciliary body, diagnosed by an ocular oncologist on multimodal imaging—B-scan ultrasonography, optical coherence tomography, wide-field fundus photography—or confirmed histopathologically.
2. Clearly documented dates for diagnosis and treatment (enucleation date or date of ruthenium-106- or iodine-125 plaque insertion).

We initially identified 380 patients with either a diagnosis date or a treatment date. Forty-four were excluded for the following specific reasons:

- **Unreliable diagnosis date (*n*** **= 21)**: for example, the operating note recorded the treatment date, but the diagnosis date was missing, derived only from second-hand correspondence, or later questioned at multidisciplinary review.
- **Unreliable treatment date (*n*** **= 19)**: typical scenarios were eyes enucleated at a referring clinic and sent to our pathology laboratory without a definitive surgery date—only the referral date, which is usually but not invariably the day of surgery, was available.
- **Misclassification (*n*** **= 2)**: originally coded as uveal melanoma but found to be choroidal metastases on re-examination of pathology and registry entries.
- **Iris melanoma (*n*** **= 2).**

After these exclusions, 336 patients remained for analysis. Of these, 151 underwent ruthenium-106 plaque brachytherapy, 52 underwent iodine-125 plaque brachytherapy, and 133 underwent primary enucleation. Plaque treatments were performed exclusively at St. Erik Eye Hospital, whereas enucleations were carried out at several major Swedish ophthalmology centers; all enucleated eyes were subsequently sent to St. Erik Eye Hospital for histopathological review, ensuring uniform diagnostic criteria and follow-up. Data were collected by the first author, who was not involved in our earlier study demonstrating the prognostic impact of treatment delays [16]. Baseline tumor characteristics—including size and AJCC stage—were recorded at diagnosis and carried forward unchanged in all analyses. Causes of death were adjudicated and classified as described previously [20]. 

### Statistical analyses

**Fig. 1:**
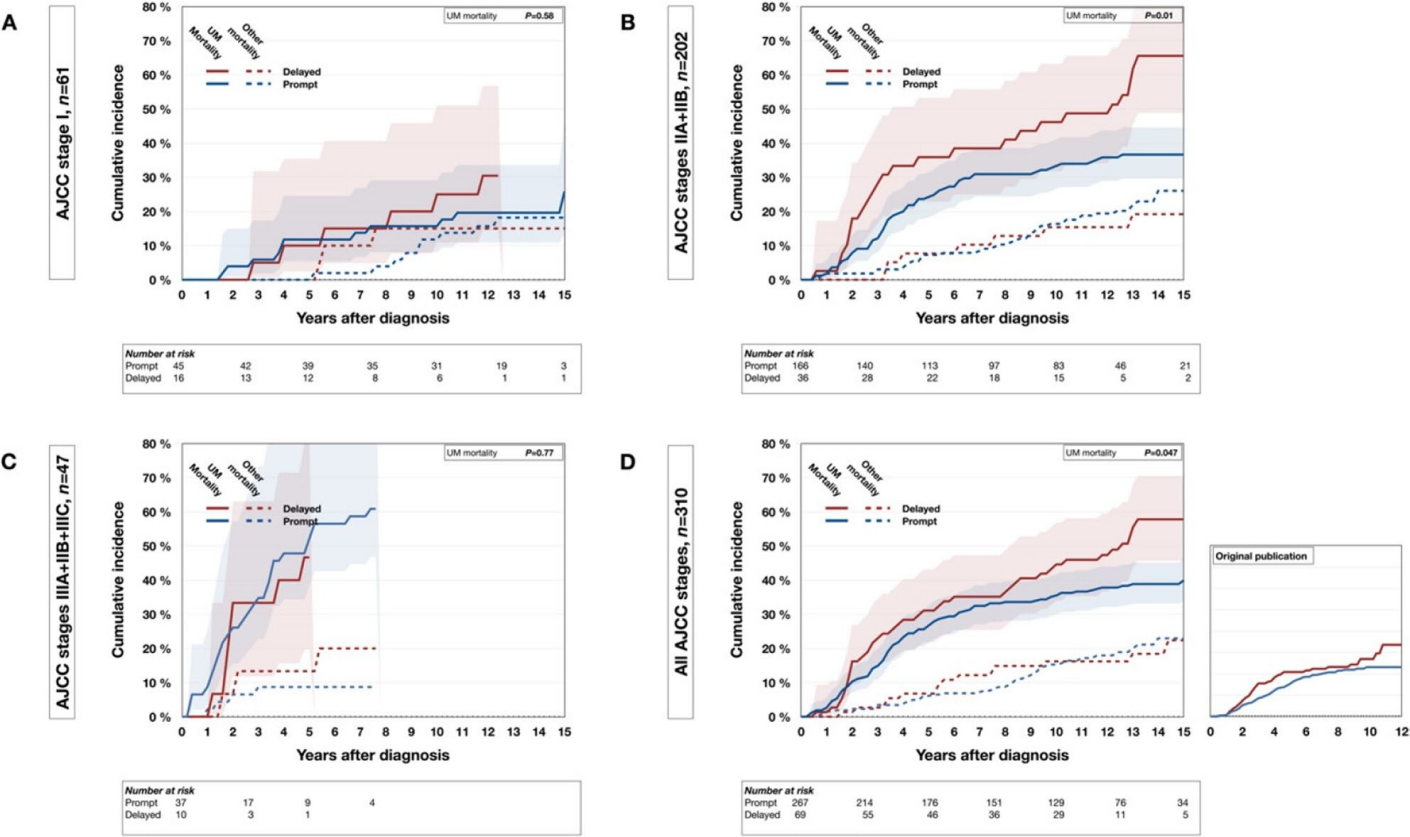
Cumulative incidence of death from metastatic uveal melanoma from competing-risk data with date of diagnosis as time zero, replicating the analysis in our original study. **A** In Stage I, as before, there was no statistically significant difference in metastatic death by treatment timing. **B** In Stage II, again, patients treated ≥ 30 days after diagnosis had a higher cumulative incidence of metastatic death. **C** In Stage III, no significant difference was observed, but the small number of patients precluded reliable plotting. **D** When Stages I–III were combined, delayed treatment (≥ 30 days) was associated with a higher cumulative incidence of metastatic death (Gray’s test, *P* = 0.047). For reference, the incidence curve from our original cohort (*n* = 1145) is inset

**Fig. 2:**
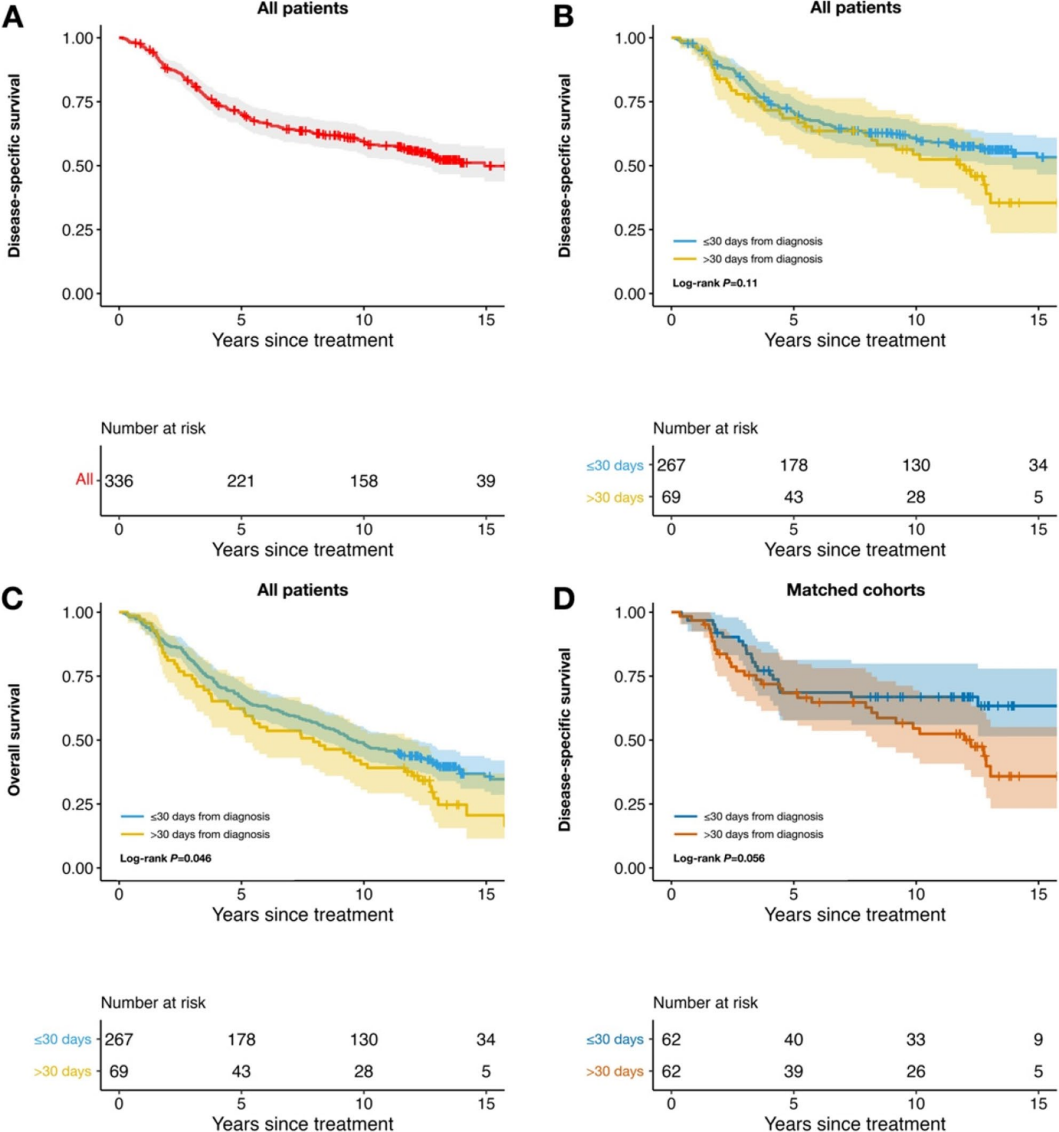
Kaplan–Meier disease-specific survival (DSS) and overall survival (OS) in patients treated ≤ 30 days versus > 30 days after diagnosis with date of treatment as time zero. **A** DSS for the entire cohort (*n* = 336), unstratified. **B** DSS for the entire cohort, stratified by treatment delay (≤ 30 vs. > 30 days). **C** OS for the entire cohort, stratified by treatment delay (≤ 30 vs. > 30 days). **D** DSS for the propensity-score–matched subgroup (*n* = 124) matched on age, largest basal tumour diameter (LBD), and tumour thickness at diagnosis. Shaded bands indicate pointwise 95% confidence intervals. *P*-values are Holm-Bonferroni corrected

**Fig. 3:**
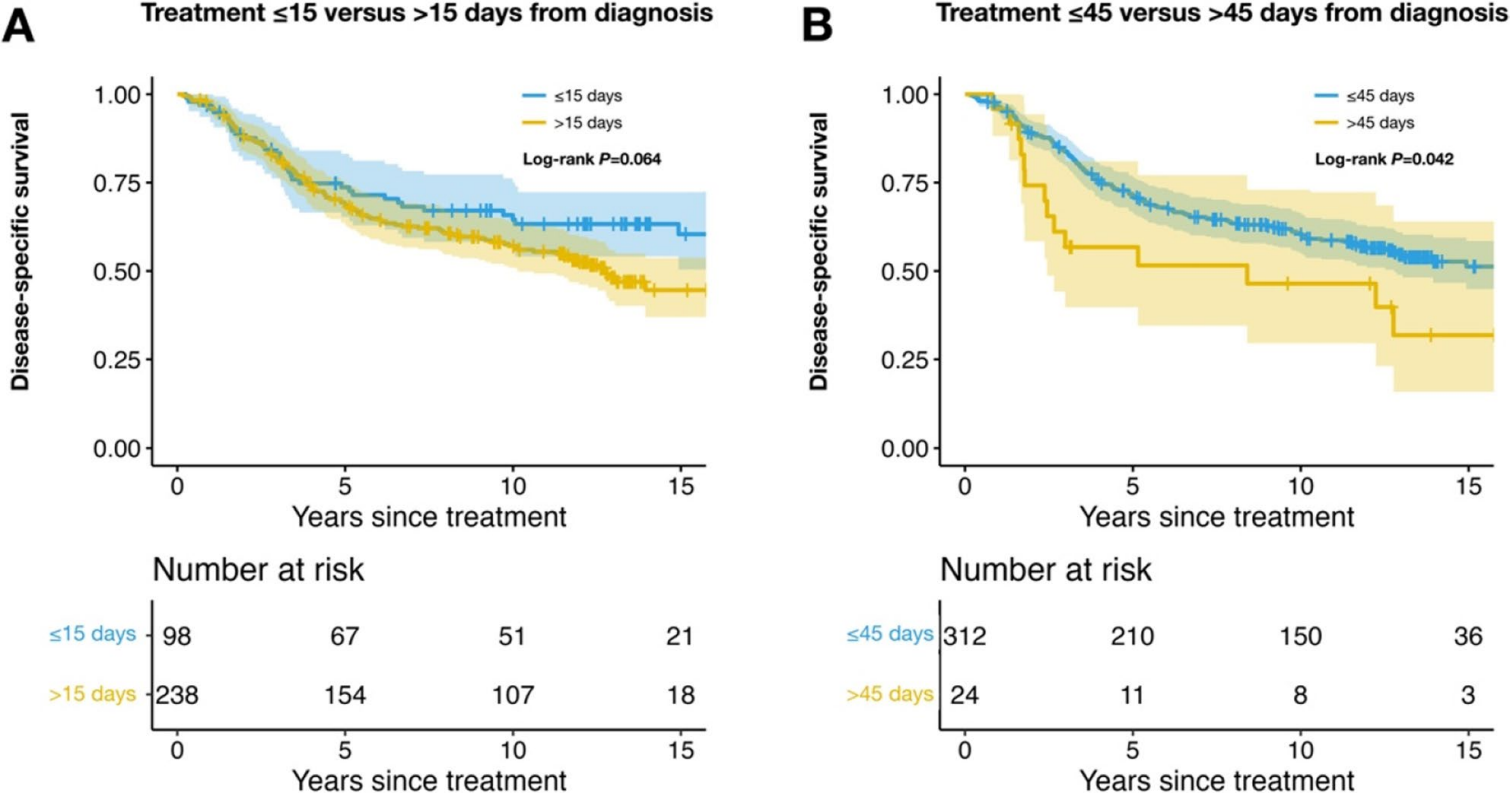
Kaplan–Meier disease-specific survival (DSS) stratified by alternative diagnosis-to-treatment cut-offs. **A** ≤ 15 days vs. > 15 days. **B** ≤ 45 days vs. > 45 days. Shaded bands indicate pointwise 95% confidence intervals. *P*-values are Holm-Bonferroni corrected

All tests were two-sided, and results were considered significant when *P* < 0.05. Holm–Bonferroni adjustment was used to control the family-wise error rate arising from multiple comparisons. Each patient was assigned a single, mutually exclusive status—alive, deceased from metastatic uveal melanoma, or deceased from other causes—and was removed from the risk set when that event occurred. Kaplan–Meier survival curves and propensity-score–matched comparisons were produced with the survival, survminer, and MatchIt packages in R (version 4.4.3; R Core Team, Vienna, 2022). Multivariable Fine–Gray competing-risks regression was performed with the crr package [21]. In our original publication, survival time was measured from the day of diagnosis. Following subsequent comments that the date of treatment may represent a preferable landmark for survival modeling, we adopted a two-step approach in the present study [8]. First, we replicated the original cumulative-incidence analyses exactly, using the day of diagnosis as time zero, to allow direct comparison with the prior report (Fig. 1). Second, for all subsequent time-to-event analyses—including Kaplan–Meier survival curves, alternative delay thresholds, propensity-score–matched analyses, and Fine–Gray competing-risk regression—the day of treatment (enucleation or plaque insertion) was used as time zero (Figs. 2 and 3, Supplementary Tables 1–4, and Supplementary Table 6).

## Results

### Descriptive statistics

Tumor size, AJCC stage, and median follow-up were comparable between patients treated within 30 days of diagnosis and those treated after 30 days. Baseline patient and tumor characteristics are summarized in Table 1, with corresponding characteristics for other delay thresholds provided in Supplementary Tables 1 to 4. The mean interval from diagnosis to treatment was 24 days (SD 19). Neither tumor thickness nor largest basal tumor diameter (LBD) was normally distributed (*P* < 0.001 and *P* = 0.004, respectively; Shapiro–Wilk test). In the Fine–Gray competing-risks model, the Grambsch–Therneau score test showed no violation of the proportional subdistribution-hazards assumption for treatment delay as a continuous variable (*P* = 0.36).

**Table 1:** Baseline characteristics of all included patients

	Treatment ≤ 30 days from diagnosis, *n* = 267	Treatment > 30 days from diagnosis, *n* = 69
Age at UM diagnosis, mean years (SD)	64 (13)	66 (13)
Tumor LBD, mean mm (SD)	12.6 (4.0)	12.4 (4.3)
Tumor thickness, mean mm (SD)	7.3 (3.7)	7.3 (3.4)
AJCC T-category, *n*(%)		
T1a	45 (18)	16 (26)
T1b	3 (1)	1 (2)
T2a	91 (37)	13 (21)
T2b	1 (< 1)	0 (0)
T3a	71 (29)	22 (36)
T3b	8 (3)	3 (5)
T3c	0 (0)	1 (2)
T4a	27 (11)	4 (7)
T4b	2 (1)	2 (3)
Na	19 (7)	7 (10)
AJCC Stage, *n*(%)		
I	45 (18)	16 (26)
IIA	94 (38)	14 (23)
IIB	72 (29)	22 (36)
IIIA	35 (14)	8 (13)
IIIB	2 (1)	2 (3)
Na	19 (7)	7 (10)
Median follow-up after treatment*, years	13.5 (12.6–21.3)	13.4 (12.4–18.5)

*AJCC *American Joint Committee on Cancer, *LBD *Largest basal diameter, *Na *missing only tumor thickness (*n*=1), only LBD (*n*=12), or both (*n*=13), precluding assignment of T‐category and AJCC stage. *SD *Standard deviation, *UM *Uveal melanoma. *Reverse Kaplan-Meier method

### Cumulative incidence

To validate our prior findings, we replicated the original cumulative-incidence analysis without modification (identical inclusion and exclusion criteria, day of diagnosis as time zero, competing-risk specification, and Gray’s test). Results closely paralleled the original publication. In Stage I, as before, there was no statistically significant difference in metastatic death by treatment timing. In Stage II, again, patients treated ≥ 30 days after diagnosis had a higher cumulative incidence of metastatic death. In Stage III, the original study detected a difference, but in the present cohort only 10 patients were treated ≥ 30 days, limiting precision and precluding reliable plotting. When Stages I–III were combined, delayed treatment (≥ 30 days) was associated with a higher cumulative incidence of metastatic death (Gray’s test, *P* = 0.047; Fig. 1).

### Kaplan–Meier survival and propensity-matched analyses at the 30-day treatment-delay threshold

Using the day of treatment as time zero, the 5-, 10-, and 15-year disease-specific survival (DSS) for the entire cohort of 336 patients were 70% (95% CI, 66–76), 59% (95% CI, 54–65), and 50% (95% CI, 44–57), respectively (Fig. 2A). Treatment begun within 30 days of diagnosis was not associated with a statistically significant DSS advantage over later treatment (Fig. 2B).

Earlier treatment was, however, associated with higher overall survival (OS). For patients treated ≤ 30 days after diagnosis, 5-, 10-, and 15-year OS were 67% (95% CI, 61–73), 48% (95% CI, 43–55), and 36% (95% CI, 30–43); corresponding values for treatment > 30 days were 62% (95% CI, 52–75), 41% (95% CI, 31–54), and 21% (95% CI, 12–37) (Fig. 2C**).**

Propensity-score matching on age, largest basal diameter, and tumor thickness produced two balanced cohorts (*n* = 62 each; Supplementary Table 5). In these matched groups, DSS again showed no meaningful difference (Fig. 2D): 5-, 10-, and 15-year DSS were 69% (95% CI, 58–81), 67% (95% CI, 56–80), and 63% (95% CI, 52–78) for treatment ≤ 30 days, versus 68% (95% CI, 58–81), 55% (95% CI, 44–69), and 36% (95% CI, 23–55) for treatment > 30 days. Early treatment therefore did not confer a statistically significant DSS benefit in either the unmatched or matched analyses at the 30-day threshold.

### Kaplan–Meier survival analyses at other treatment-delay thresholds

When a 15-day cut-off was applied, DSS remained similar to that observed with the 30-day definition of “early” treatment (Fig. 3A).

Once the threshold was extended to 45 days, a disadvantage became apparent for longer delays (Fig. 3B). Patients treated ≤ 45 days after diagnosis had 5-, 10-, and 15-year DSS of 71% (95% CI, 67–77), 60% (95% CI, 55–66), and 51% (95% CI, 45–59), respectively. The corresponding figures for those treated > 45 days were 57% (95% CI, 40–81), 46% (95% CI, 30–73), and 32% (95% CI, 16–64).

### Competing risks regression analysis

In a multivariable Fine–Gray competing-risks model, we set the day of treatment as the time origin, considered death from other causes as a competing event, and adjusted for patient age and AJCC stage. Each 10-day increase from diagnosis to treatment was associated with a 15% higher subdistribution hazard (sHR 1.16; 95% CI, 1.10–1.22; Supplementary Table 6). To test whether delay could be modeled as a linear covariate, we compared linear, quadratic, and restricted cubic spline specifications. Neither the quadratic term nor spline models provided better fit than the linear model (ΔAIC < 2, likelihood ratio *P* = 0.64), supporting the use of a continuous linear term for delay.

### Modelled temporal evolution of choroidal melanoma volume and associated 10-year metastatic mortality risk

**Fig. 4:**
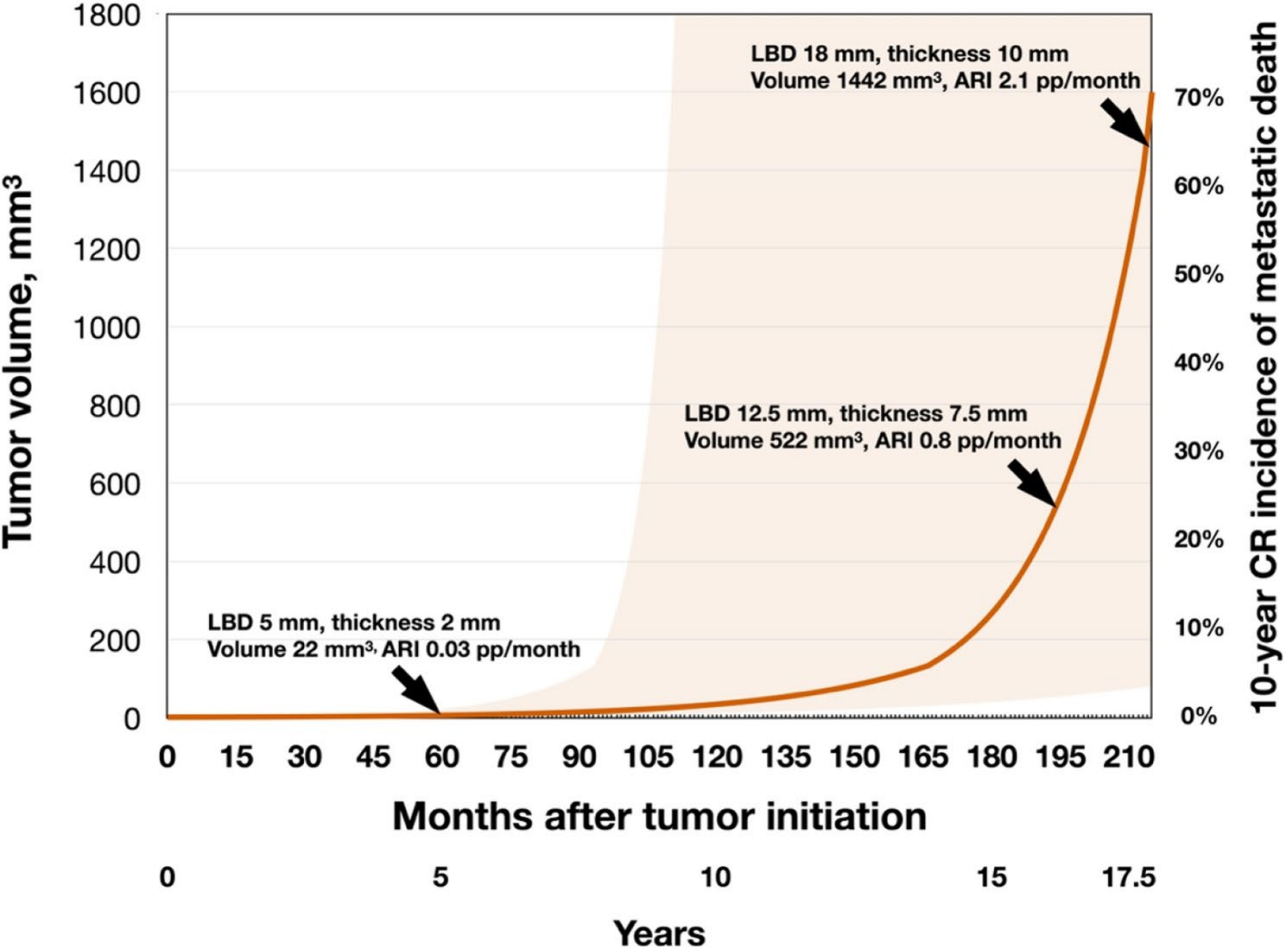
Modeled temporal evolution of choroidal melanoma volume and the corresponding 10-year metastatic-mortality risk. The volume-growth curve starts at a reference volume of 1 mm³ and follows exponential expansion based on published volume-doubling times for tumours classified as small, medium, or large under the revised Collaborative Ocular Melanoma Study (COMS) criteria [19, 22–24]. For a different starting volume (e.g., 0.5 mm³), add ~ 2 years to the time axis, assuming a doubling time of 717 days (95% confidence interval (CI), 401–1033) for small melanomas. The secondary (right) y-axis displays the modeled 10-year competing-risks incidence of death from metastatic uveal melanoma derived from volume-based estimates [11]. The mean lesion in the present cohort (largest basal diameter (LBD) 12.5 mm; apical thickness 7.5 mm) maps to 522 mm^3^ on the curve. With a volume-doubling time of 421 days (95% CI, 145–700 days), the tumour is predicted to reach 547 mm^3^ after 1 month, raising the modeled 10-year competing-risks incidence from 21.49% to 22.24%—an absolute risk increase (ARI) of 0.8% points. If a small tumour (LBD 5 mm; thickness 2 mm; estimated volume 22 mm^3^) remains untreated for 1 month, the ARI is 0.03% points. For a large tumour (LBD 18 mm; thickness 10 mm; estimated volume 1442 mm^3^) left untreated for the same period, the ARI is 2.1% points. The red shaded area represents the 95% CI. For individual patients, these estimates should be adjusted for additional prognostic factors beyond tumour size, including genetic status

The average tumor in this cohort had a largest basal diameter of 12.5 mm and a thickness of 7.5 mm, corresponding to an estimated volume of 522 mm^3^ [11]. Exponential doubling times derived from the revised Collaborative Ocular Melanoma Study (COMS) criteria are 717 days (95% CI, 410–1033) for small tumors, 421 days (95%CI, 145–700) for medium tumors, and 307 days (95% CI, 143–471) for large tumors [19]. Applying these values shows that a 522 mm^3^ tumor increases by roughly 25 mm^3^ per month at its current position on the exponential growth curve (Fig. 4). Published competing-risk data relating tumor volume to 10-year melanoma-specific mortality suggest an estimated absolute increase of 0.8% points over the preceding month [11]. 

## Discussion

This study confirms that longer intervals between diagnosis and definitive treatment of posterior uveal melanoma are associated with progressively poorer survival. This relationship persisted in cumulative incidence analysis, propensity-matched Kaplan–Meier curves and in Fine–Gray competing-risk regression analyses, which—unlike in our earlier work—used the day of treatment as time zero. Overall, the prognostic impact of a prolonged interval between diagnosis and treatment was similar in this cohort to that observed in our previous cohort of non-overlapping patients [16]. These findings are also consistent with the results we reported in a systematic review and meta-analysis based on tumor doubling time data and prognostic correlations from several independent institutions [19]. 

Our aim was to determine whether longer delay is associated with a higher risk of metastatic death, not to argue for a particular effect size. Still, the magnitude is informative: in multivariable Fine–Gray regression, each 10-day increase in delay was associated with an sHR of 1.16, similar to the sHR per millimeter increase in tumor diameter (1.13)—a factor that is widely recognized as one of the strongest and most consistent prognostic markers in uveal melanoma. At the same time, AJCC stage was a far stronger marker in our data. We did not have chromosomal or genetic information in this cohort, which is undoubtedly a limitation. Cytogenetic and genetic alterations (e.g., chromosome 3 loss, *BAP1* mutation) are almost certainly more important determinants of outcome than the interval between diagnosis and treatment. Nonetheless, clarifying whether treatment delay independently associates with metastatic death matters for both biology and care pathways. If such an association exists, ocular treatment may prevent some metastatic deaths—implying that intervening on the primary tumor can modify the disease course—and it can inform hypotheses about the timing and sequence of metastatic seeding at chromosomal and genetic levels.

Importantly, we do not question the observation that much of a uveal melanoma’s metastatic potential is determined early in tumor progression [25]. For instance, tumors with *EIF1AX* mutations typically carry a low metastatic risk, even if they reach a large size. Our findings do not contradict this principle but rather suggest a difference in degree: if tumors are left to grow, some—though not all—will acquire additional aggressive traits that affect prognosis. While it is often assumed that high-risk features like monosomy 3 are present from inception, evidence suggests that genomic evolution can occur later in the disease course. Indeed, the prevalence of monosomy 3 and 8q gains increases with tumor size, rising from < 25% in the smallest tumors to > 70% in the largest [11, 26]. This is exemplified by documented cases where tumors appeared quiescent for years before undergoing sudden expansion; in such instances, the newer, rapidly growing areas exhibited monosomy 3 and chromosome 8q gains, while the older basal portions remained disomy 3 [27]. Such observations support the hypothesis that late transformation to a highly malignant genotype can occur during observation, significantly increasing the risk of metastatic spread. This perspective is further supported by evidence that primary tumor size is associated with both survival time in metastatic disease and the burden of metastatic lesions at detection—both of which suggest a potential survival benefit to treating the primary tumor [3, 14, 28, 29]. 

Notably, Straatsma et al. (2003) reported a Kaplan–Meier estimate of 5-year mortality of 30% (95% CI, 18–47) for patients who deferred, or received no, primary tumor treatment, compared with 18% (95% CI, 16–20) for those treated promptly; the adjusted risk ratio, however, did not reach statistical significance [30]. The authors provided no *P* values for their time-to-event analyses, and a later re-analysis by our group indicates that the survival curves were significantly separated after all [31]. 

Considering that the effect size inevitably depends on cohort size and analytical method, it is unsurprising that the present, smaller cohort (*n* = 336) showed significant divergence for DSS only when treatment was delayed beyond 45 days, whereas our prior series of 1145 patients revealed a difference at 30 days. Lower statistical power is the most plausible explanation, and it should also be pointed out that herein, survival was analyzed with Kaplan–Meier plots rather than cumulative-incidence curves to address previous methodological criticism.

The detriment associated with delay is not explained by larger tumors at baseline: baseline AJCC stage was included in the multivariate competing-risk model yet delay still retained significance. In fact, in the earlier cohort tumors were larger at diagnosis among patients treated within 30 days [16]. These observations suggest that what matters is tumor size—and biological behavior—up to the moment of treatment. A prolonged interval could allow for continued growth, acquisition of additional cytogenetic abnormalities, and extended shedding of malignant cells.

Experimental and clinical evidence supports ongoing dissemination until the primary lesion is eradicated. We and others have detected micrometastases in most patients and circulating tumor cells even when lesions are small [32, 33]. Later-arising metastases frequently bear extra genetic aberrations and may progress more aggressively [29, 34]. Delaying treatment beyond roughly six weeks is therefore potentially linked to a higher probability of lethal clone seeding.

All lesions analyzed here had already been diagnosed as melanoma, sometimes after an initial observation period. Accordingly, these findings are not applicable to nevi or indeterminate lesions, where observation until diagnostic certainty is achieved remains appropriate. Once a diagnosis of melanoma has been established, however, the data support initiating treatment within 30 to 45 days and advise against extended observation after diagnosis.

### Strengths and limitations

This study reaffirms the prognostic importance of the time to treatment of uveal melanoma, drawing on a substantial dataset from the only Swedish national referral center. The completeness of follow-up data bolsters the reliability of our findings, providing valuable insights into an area still under debate. Furthermore, our application of multiple statistical approaches supports the observed association between time to treatment and survival outcomes, indicating that these findings are not merely artifacts of any single methodological choice.

Several limitations merit consideration. First, the lack of a statistically significant difference in DSS at certain thresholds (e.g., 30 days) and in matched analyses likely reflects limited statistical power compared to our previously published larger cohort [16]. Small sample sizes in specific subgroups, such as Stage III patients with long delays, further restrict our ability to detect smaller effect sizes. This applies in particular to stratified and matched analyses, where effective sample size is reduced by design. In addition, we applied Holm–Bonferroni adjustment to control the family-wise error rate across multiple prespecified comparisons. This conservative approach reduces the probability of false-positive findings but can decrease statistical power, particularly in smaller samples. Accordingly, borderline results after correction should be interpreted cautiously, with emphasis on effect sizes and confidence intervals rather than dichotomous significance thresholds.

Second, disease-specific outcomes were evaluated using both cumulative-incidence methods and Kaplan–Meier curves, which address different estimands in the presence of competing events. Cumulative-incidence analysis with Gray’s test explicitly accounts for death from other causes, whereas Kaplan–Meier estimation treats competing deaths as non-informative censoring. In a cohort with substantial non-melanoma mortality, these methodological differences can yield divergent apparent separations for melanoma-specific outcomes and should be interpreted accordingly.

Third, the retrospective, non-randomized design leaves room for unmeasured confounding; patients who waited longer may have differed in ways not captured by available variables. A prospective randomized trial would address this problem, but withholding prompt treatment raises obvious ethical concerns.

Fourth, although the cohort was collected independently of our earlier report, this validation was performed at the same referral center with overlapping investigators. While this continuity may limit independence, it also ensured consistent diagnostic criteria and inclusion rules. Importantly, data collection for the current study was conducted by the first author, who was not involved in the previous analysis, reducing the risk of overlap or bias.

Fifth, cause of death was determined from Swedish death certificates, which list an underlying cause of death and up to 15 contributory diagnoses in the causal chain. We classified metastatic uveal melanoma as the underlying cause when metastatic disease was recorded among the causative diagnoses leading to death, even if the terminal event was organ failure or circulatory insufficiency. Nonetheless, death-certificate–based classification is imperfect, and misclassification (either omission or miscoding of metastatic disease) could still have attenuated or exaggerated the observed association with treatment delay.

Sixth, we lacked genetic and cytogenetic data and re-measured tumor dimensions infrequently at the time of treatment, preventing a direct link between interval length, interim tumor growth, and molecular risk factors. Future work should include periodic imaging and molecular profiling to clarify these relationships.

Seventh, tumor volumes were estimated assuming a semi-ellipsoid shape. This may not reflect every lesion, and ellipsoid models may overestimate volume compared with contrast-enhanced T1-weighted MRI [35]. However, photos, ultrasound, and OCT may show greater tumor extent than MRI, and, more importantly, as long as the same input data (LBD and thickness) are used, doubling times remain identical regardless of whether an ellipsoid, cubical, cylindrical, or other shape is assumed [11, 19]. 

Finally, using the day of treatment as time zero could introduce time-to-treatment initiation bias, which can shorten observed survival in delayed cases. However, our earlier study, which measured survival from the day of diagnosis, produced similar results, suggesting that this bias did not meaningfully distort the present findings [16]. 

## Conclusions

Our findings suggest that longer intervals between diagnosis and treatment may be associated with poorer outcomes in uveal melanoma. While observation for growth may be necessary for diagnostic purposes—and exceptions exist, including patients with small tumors, monocular patients, and those with very limited life expectancy—our data support initiating treatment within 30–45 days once a melanoma diagnosis is confirmed.

## Supplementary Information

Supplementary Material 1.

## Data Availability

Patient-level data for this study are available at [https://rcsyd.se](https:/rcsyd.se) . Approval from the Swedish Ethical Review Authority is required before access can be granted.
