# Supplementary material for: Independent validation of time to treatment as a prognostic factor in uveal melanoma
Source: BMC Cancer. 2026 Feb 19;26:302. doi: 10.1186/s12885-026-15775-z (PMC12930993; doi:10.1186/s12885-026-15775-z)
Supplement: Supplementary file 1 — Supplementary Material 1. [file 12885_2026_15775_MOESM1_ESM.pdf]

Supplementary material

Independent validation of time to treatment as a prognostic factor in uveal melanoma

Annahita Moghadam, M.D.[1], Viktor Torgny Gill, M.D.[1,2], Shiva Sabazade, M.D.[1,3], Anna Hagström, M.D.[1], Gustav Stålhammar, M.D. Ph.D.[1,3]

[1]Department of Clinical Neuroscience, Division of Eye and Vision, Karolinska Institutet, Stockholm, Sweden
[2]Department of Pathology, Västmanland Hospital Västerås, Västerås, Sweden
[3]St. Erik Eye Hospital, Stockholm, Sweden

Table of contents

SUPPLEMENTARY TABLE 1 2
SUPPLEMENTARY TABLE 2 3
SUPPLEMENTARY TABLE 3 4
SUPPLEMENTARY TABLE 4 5
SUPPLEMENTARY TABLE 5 6
SUPPLEMENTARY TABLE 6 7

**15-day** threshold for early vs. late treatment	Treatment ≤15 days from diagnosis, n=98	Treatment >15 days from diagnosis, n=238
Age at UM diagnosis, mean years (SD)	61 (14)	65 (13)
Tumor LBD, mean mm (SD)	12.7 (4.5)	12.6 (3.9)
Tumor thickness, mean mm (SD)	7.8 (3.8)	7.1 (3.5)
AJCC T-category, n (%)		
T1a	20 (20)	41 (17)
T1b	0 (0)	4 (2)
T2a	23 (23)	81 (34)
T2b	0 (0)	1 (<1)
T3a	28 (29)	65 (27)
T3b	4 (4)	7 (3)
T3c	0 (0)	1 (<1)
T4a	13 (13)	18 (8)
T4b	0 (0)	4 (2)
Na	10 (10)	16 (7)
AJCC Stage, n (%)		
I	20 (20)	41 (17)
IIA	23 (23)	85 (36)
IIB	28 (29)	66 (28)
IIIA	17 (17)	26 (11)
IIIB	0 (0)	4 (2)
Na	10 (10)	16 (7)
Median follow-up after treatment*, years	15.1 (13.2–26.9)	13.1 (12.3–18.5)

Supplementary Table 1
Baseline characteristics of patients grouped by a 15-day threshold for early versus late treatment. For comparison, Table 1 in the main manuscript shows the corresponding characteristics at the 30-day threshold.

**45-day** threshold for early vs. late treatment	Treatment ≤45 days from diagnosis, n=312	Treatment >45 days from diagnosis, n=24
Age at UM diagnosis, mean years (SD)	64 (13)	70 (14)
Tumor LBD, mean mm (SD)	12.6 (4.0)	12.7 (4.9)
Tumor thickness, mean mm (SD)	7.2 (3.6)	7.9 (3.3)
AJCC T-category, n (%)		
T1a	57 (18)	4 (17)
T1b	3 (<1)	1 (4)
T2a	100 (32)	4 (17)
T2b	1 (<1)	0 (0)
T3a	84 (27)	9 (38)
T3b	10 (3)	1 (4)
T3c	1 (<1)	0 (0)
T4a	29 (9)	2 (8)
T4b	3 (<1)	1 (4)
Na	24 (8)	2 (8)
AJCC Stage, n (%)		
I	57 (18)	4 (17)
IIA	103 (33)	5 (21)
IIB	85 (27)	9 (38)
IIIA	40 (13)	3 (12)
IIIB	3 (<1)	1 (4)
Na	24 (8)	2 (8)
Median follow-up after treatment*, years	13.4 (12.4–21.3)	18.5 (13.9–37.1)

Supplementary Table 2
Baseline characteristics of patients grouped by a 45-day threshold for early versus late treatment.

**60-day** threshold for early vs. late treatment	Treatment ≤60 days from diagnosis, n=329	Treatment >60 days from diagnosis, n=7
Age at UM diagnosis, mean years (SD)	64 (13)	72 (13)
Tumor LBD, mean mm (SD)	12.6 (4)	12.3 (5.6)
Tumor thickness, mean mm (SD)	7.3 (3.6)	6.6 (3.5)
AJCC T-category, n (%)		
T1a	60 (18)	1 (14)
T1b	3 (<1)	1 (14)
T2a	103 (31)	1 (14)
T2b	1 (<1)	0 (0)
T3a	90 (27)	3 (43)
T3b	10 (3)	1 (14)
T3c	1 (<1)	0 (0)
T4a	31 (9)	0 (0)
T4b	4 (1)	0 (0)
Na	26 (8)	0 (0)
AJCC Stage, n (%)		
I	60 (18)	1 (14)
IIA	106 (32)	2 (29)
IIB	91 (28)	3 (43)
IIIA	42 (13)	1 (14)
IIIB	4 (1)	0 (0)
Na	26 (8)	0 (0)
Median follow-up after treatment*, years	13.5 (12.4–21.3)	NA

Supplementary Table 3
Baseline characteristics of patients grouped by a 60-day threshold for early versus late treatment.

**90-day** threshold for early vs. late treatment	Treatment ≤90 days from diagnosis, n=334	Treatment >90 days from diagnosis, n=2
Age at UM diagnosis, mean years (SD)	64 (13)	78 (12)
Tumor LBD, mean mm (SD)	12.6 (4.1)	17 (1.4)
Tumor thickness, mean mm (SD)	7.3 (3.6)	6 (2.8)
AJCC T-category, n (%)		
T1a	61 (18)	0 (0)
T1b	4 (1)	0 (0)
T2a	104 (31)	0 (0)
T2b	1 (<1)	0 (0)
T3a	91 (27)	2 (100)
T3b	11 (3)	0 (0)
T3c	1 (<1)	0 (0)
T4a	31 (9)	0 (0)
T4b	4 (1)	0 (0)
Na	26 (8)	0 (0)
AJCC Stage, n (%)		
I	61 (18)	0 (0)
IIA	108 (32)	0 (0)
IIB	92 (28)	2 (100)
IIIA	43 (13)	0 (0)
IIIB	4 (1)	0 (0)
Na	26 (8)	0 (0)
Median follow-up after treatment*, years	13.5 (12.4–21.3)	NA

AJCC, American Joint Committee on Cancer. LBD, largest basal diameter. Na, missing tumor thickness or LBD data, precluding assignment of T-category and AJCC stage. SD, standard deviation. UM, uveal melanoma.

*Reverse Kaplan–Meier method.

Supplementary Table 4
Baseline characteristics of patients grouped by a 90-day threshold for early versus late treatment.

Supplementary Table 5

Baseline characteristics of matched cohorts

	Treatment ≤30 days from diagnosis, *n*=62	Treatment >30 days from diagnosis, *n=62*
Age at UM diagnosis, mean years (SD)	66 (12)	66 (13)
Tumor LBD, mean mm (SD)	12.2 (3.9)	12.3 (4.3)
Tumor thickness, mean mm (SD)	7.1 (3.6)	7.2 (3.4)
AJCC T-category, *n* (%)		
T1a	12 (19)	16 (26)
T1b	0 (0)	1 (2)
T2a	23 (37)	13 (21)
T3a	20 (32)	22 (36)
T3b	2 (3)	3 (5)
T3c	0 (0)	1 (2)
T4a	5 (8)	4 (7)
T4b	0 (0)	2 (3)
AJCC Stage, *n* (%)		
I	12 (19)	16 (26)
IIA	23 (37)	14 (23)
IIB	20 (32)	22 (36)
IIIA	7 (11)	8 (13)
IIIB	0 (0)	2 (3)
Median follow-up after treatment*, years	13.9 (12.1–27.8)	13.8 (12.4–37.1)

AJCC, American Joint Committee on Cancer. LBD, largest basal diameter. SD, standard deviation. UM, uveal melanoma. *Reverse Kaplan-Meier method.

Supplementary Table 6

Multivariate Fine–Gray competing risks regression: subdistribution hazard ratios (exp β) for death from metastatic uveal melanoma, treating death from other causes as a competing event.

	β_j	S.E.	*z*	*P*	exp(β_j)	95 % CI
**Multivariate**						
Interval[a]	0.14	0.03	5.3	<0.001	1.16	1.10 to 1.22
Patient age at diagnosis[b]	0.01	0.01	0.3	0.76	1.00	0.99 to 1.02
LBD	0.12	0.03	4.4	<0.001	1.13	1.07 to 1.19
Tumor thickness	0.02	0.03	0.9	0.37	1.03	0.97 to 1.09

Day of treatment set as time origin. [a]Per 10-day increase in time from diagnosis to treatment. [b]Per increasing year. [c]Per one-unit increase in AJCC stage at the time of diagnosis (I → IIA → IIB → IIIA → IIIB → IIIC). AJCC, American Joint Committee on Cancer; LBD, largest basal tumor diameter; S.E., standard error; *z*, Wald Z statistic.
